# Epidemiology of Hepatitis B Virus Infection in Nigeria: A Narrative Review of Prevalence, Transmission, Genotypes, Coinfections, and Mortality

**DOI:** 10.1155/jotm/4939367

**Published:** 2025-09-22

**Authors:** Babayemi O. Olakunde, Daniel A. Adeyinka, Olubunmi A. Olakunde, Stanley C. Eneh, Temitayo Ogundipe

**Affiliations:** ^1^Department of Population and Community Health, College of Public Health, University of North Texas Health Science Center, Fort Worth, Texas, USA; ^2^IVAN Research Institute, University of Nigeria, Nsukka, Enugu, Nigeria; ^3^Department of Research, Saskatchewan Health Authority, Saskatoon, Saskatchewan, Canada; ^4^National AIDS and STI Control Programme, Department of Public Health, Federal Ministry of Health, Abuja, Nigeria; ^5^Department of Disease Control and Immunization, Ondo State Primary Health Care Development Agency, Akure, Ondo, Nigeria; ^6^Department of Hospital Medicine, Sentara Williamsburg Regional Medical Center, Williamsburg, Virginia, USA

## Abstract

**Background:**

Nigeria has the highest burden of hepatitis B virus (HBV) infection in sub-Saharan Africa. However, the lack of a robust surveillance system and program data has limited the understanding of the burden and the distribution of HBV across different populations. This narrative review aimed to summarize available data on the epidemiology of HBV in Nigeria and identify research gaps in the existing literature.

**Methods:**

We searched PubMed, Scopus, and Google Scholar for relevant articles published between January 2000 and June 2025. Primary studies, reviews, and reports that contained data of interest, including prevalence, incidence, mode of transmission, mortality, and genotypes, were included in this review. Where available, we restricted our results to findings from representative surveys (conducted across the six geopolitical zones) or systematic reviews. Prevalence rates < 2%, 2%–7%, and ≥ 8% were described as low, intermediate, and high, respectively.

**Results:**

Studies on the prevalence of hepatitis B surface antigen (HBsAg) have reported intermediate to high rates in the general population (5.4%–13.6%), with evidence suggesting declining trend. The most recent estimates showed a prevalence of 5.4% in 2022, corresponding to approximately 14.4 million people living with HBV. Available data indicate sociodemographic disparities in HBV prevalence, with higher rates among men, adults (> 18 years), and rural dwellers. Reported prevalence rates among specific subpopulations include blood donors (13.2%–14.0%), pregnant women (5.5%–14.1%), prison inmates (4%–42.2%), people who inject drugs (7%–7.8%), healthcare workers (1.1%–25.7%), female sex workers (0%–17.1%), men who have sex with men (8.4%–11.7%), and transgender women (15.6%). The prevalence of hepatitis B e-antigen (HBeAg) in the general population ranged from 6.0% to 23.6%. The prevalence of occult HBV infection (OBI) ranged from 0.9% to 17.0%. Genotype E was consistently reported as the predominant HBV genotype. Most studies reported low, intermediate, and high prevalence rates for HBV-hepatitis C virus (HCV), HBV-hepatitis D virus (HDV), and HBV-HIV coinfections, respectively. In 2022, approximately 46,000 deaths were attributed to HBV, translating to a mortality rate of 21 per 100,000 population.

**Conclusions:**

A wide range of HBV prevalence rates has been observed across various population groups in Nigeria. Key research gaps in HBV epidemiology that must be addressed include modes of transmission, incidence rate, prevalence among key populations, prevalence of OBI in the general population, and spatial distribution of the burden.

## 1. Background

Despite the availability of effective vaccines for the prevention of hepatitis B virus (HBV) infection and substantial advances in antiviral treatments, HBV remains a major global public health problem, particularly in sub-Saharan Africa (SSA) [1, 2]. An estimated 771,000 people are newly infected with HBV each year, and approximately 272,000 die from it annually in SSA [3]. Although the global plan to eliminate HBV has gained momentum, emerging evidence suggests that many countries in SSA are not on track to achieve the 2030 targets of a 90% reduction in new infections and a 65% reduction in HBV-related mortality [4].

Nigeria bears the highest burden of HBV in SSA [3], and a significant proportion of the individuals living with the virus remain undiagnosed and untreated [3, 5]. In response, the country has committed to eliminating HBV as a public health threat by 2030 [6, 7]. The 2022 National Strategic Framework outlines several strategic actions, including the delivery of people-centered, evidence-based services and the use of data to improve access to prevention and treatment services for viral hepatitis, in pursuit of this ambitious yet attainable goal [6]. Progress toward these commitments, however, has been slow. Nigeria remains among the countries facing unique challenges in the prevention and control of viral hepatitis [3].

As in many other resource-limited settings, the absence of a robust surveillance system and limited programmatic data in Nigeria [8] have constrained a comprehensive understanding of the burden of HBV and impeded the implementation of effective prevention and treatment strategies. However, this gap is increasingly being bridged through the availability of national surveys. The Nigeria HIV/AIDS Indicator and Impact Survey (NAIIS), conducted in 2018, represented the largest national household-based survey to assess the prevalence of HBV, providing critical insights into its distribution within the general population [9]. Additionally, the number of systematic reviews and empirical studies examining prevalence of HBV among specific subpopulations has grown in recent years.

The ongoing efforts to eliminate HBV in Nigeria require a comprehensive understanding of its epidemiological patterns. Accordingly, this study aims to review and synthesize existing data on the epidemiology of HBV in Nigeria and to identify key knowledge gaps in the current literature.

## 2. Methods

We searched PubMed, Scopus, and Google Scholar for relevant articles published between January 2000 and June 2025 using search terms relating to HBV, Epidemiology, and Genotype and individual names of the states in Nigeria. The references of articles obtained were reviewed for additional relevant articles. In addition, we searched Google for gray literature. Primary studies, reviews, reports, and preprints that contained data of interest, including prevalence, incidence, mode of transmission, mortality, and genotypes, were included in this review. Primary studies with sample size of less than 100 were excluded. Where available, we restricted our results to findings from representative surveys (conducted across the six geopolitical zones) or systematic reviews. For the purpose of this study, we described prevalence ≥ 8% as high, 2%–7% as intermediate, and < 2% as low [10, 11].

## 3. Results

### 3.1. Prevalence of HBV

Studies on the prevalence of hepatitis B surface antigen (HBsAg+) have reported high to intermediate prevalence rates among the general population in Nigeria (Table 1). The first meta-analysis on the prevalence of HBV in Nigeria, which included 34,376 persons from 46 studies published between 2000 and 2013, reported a prevalence rate of 13.6% [12]. However, lower rates have been reported subsequently. For example, the NAIIS, a nationally representative household survey, reported a prevalence of 8.1% among adults aged 15–64 years. A more recent meta-analysis of 47 studies published between 2010 and 2019, involving 21,702 participants, found a pooled prevalence of 9.5% [13]. The Global Burden of Disease (GBD) also estimated a prevalence of 9.9% in 2019 [14]. Recent data from the World Health Organization (WHO) indicated an intermediate endemicity of HBV in Nigeria, with an estimated prevalence of 5.4% in 2022 [3]. Collectively, these studies suggest a downward trend in the prevalence of HBV in Nigeria. Indeed, a meta-regression analysis by Musa et al. showed an annual decline of 0.8% in HBV prevalence [12]. Data from the GBD also indicated a decrease in the prevalence of HBV in Nigeria, from 10.6% in 2015 to 9.9% in 2019 [14], translating to an average annual percentage decline of 1.81%.

### 3.2. Sociodemographic Disparities in HBV Prevalence

#### 3.2.1. Gender

Evidence suggests gender disparity in the prevalence of HBV in Nigeria. In a survey of 5558 adults across the country's six geopolitical regions, Onyekwere and Hameed reported a prevalence of 8.1% in men, which was more than twice that of women (3.2%) [47]. This pattern was corroborated by a national study conducted by Olayinka et al., which found a prevalence of 14.5% in men compared with 8.5% in women [48]. Similar disparity was also observed in NAIIS, with a prevalence of 10.3% in men and 5.8% in women [9].

#### 3.2.2. Age

Two systematic reviews have reported a higher prevalence of HBV in adults compared with children. Musa et al. found a prevalence of 11.5% in children (≤ 12 years) and 14.0% in adults (≥ 18 years) [12]. Although Ajuwon and colleagues used a different age categorization, their findings were consistent, with a prevalence of 11.4% in children (≤ 17 years) versus 12.7% in adults (> 17 years) [13]. NAIIS provided further insights by age bands, with the prevalence highest among persons 35–39 years (10.2%) and lowest among those aged 55–59 years (2.5%) [9].

#### 3.2.3. Area of Residence

Available data suggest regional differences in the prevalence of HBV, with the northern region having a higher burden than the southern region. For example, Musa et al. reported a prevalence of 14.7% in the northern region compared with 13.6% in the southern region [12]. In addition to regional variation, studies have also shown that HBV prevalence is higher among rural dwellers. A review by Ajuwon et al. reported a prevalence of 10.7% among rural populations, compared with 8.2% in urban areas [13]. Similarly, data from NAIIS showed a slightly higher prevalence in rural areas (8.5%) than in urban areas (7.6%) [9].

#### 3.2.4. Education

Findings on the prevalence of HBV by educational attainment are mixed and inconclusive. While some studies have reported a slightly higher prevalence among more educated persons, others have found the opposite. For instance, in NAIIS, the prevalence of HBV among people with tertiary education (8.6%) was slightly higher than those with primary education (7.7%) and no formal education (7.2%) [9]. Conversely, Olayinka et al. found a higher prevalence of HBV among individuals with no formal education (13.1%) compared with those with tertiary education (12.1%) [48].

#### 3.2.5. Economic Status

Studies examining the prevalence of HBV by economic status have considered both wealth and income, but the findings remain inconsistent. The NAIIS reported HBV prevalence by wealth quintile, showing lower prevalence in higher wealth groups: 0.1% and 0.2% in the highest and fourth quintiles, respectively, compared with 1.9% and 2.0% in the lowest and second quintiles [9]. In contrast, Olayinka et al. reported a different pattern based on monthly income. Their study found a lower prevalence (11.4%) among individuals earning less than ₦18,000, compared with 13.9% and 13.1% among those earning ₦18,000 to  ₦34,999 and ≥₦35,000, respectively [48].

### 3.3. HBV Prevalence in Specific Subpopulations

#### 3.3.1. Blood Donors

HBV is reportedly the most prevalent transfusion-transmissible infection among blood donors in Nigeria [49, 50]. Available systematic reviews consistently demonstrated a high prevalence of HBV within this population. For instance, Musa et al. reported a prevalence of 14.0% among blood donors, slightly higher than 13.6% observed in the general population [12]. Similarly, Ajuwon et al. found a greater difference, with a prevalence of 13.2% among blood donors compared with 9.5% in the general population [13].

#### 3.3.2. Key Populations

The majority of studies on key populations (persons at increased risk of HBV due to high-risk behaviors) have predominately focused on incarcerated persons, with limited data available on other groups, particularly men who have sex with men, people who inject drug, and transgender population (Table 1). Studies among inmates have reported a wide range of HBV prevalence, from 4% to 42.2% (median: 15.5%) [15–24], with most indicating high prevalence in this population (Table 1). Similarly, studies among female sex workers (FSWs) have reported a range of 0%–17.1% (median: 6%) [27–30]. Among PWID, studies in Lagos and Enugu States found HBV prevalence of 7.8% [32] and 7%, respectively [31]. Two studies conducted among MSM reported HBV prevalence of 11.7% [25] and 8.4% [26], while an available study among transgender women found a prevalence of 15.6% [26].

#### 3.3.3. Pregnant Women

In the systematic reviews by Musa et al. and Ajuwon et al., the subgroup analyses showed a high prevalence of HBV among pregnant women, at 14.1% [12] and 7.7% [13], respectively. A more specific systematic review that included 26,548 pregnant women across 20 studies published between 2014 and 2021 estimated a pooled prevalence of HBV among pregnant women as 6.5% [46]. Similarly, intermediate prevalence of 5.5% was reported among pregnant women in NAIIS [9].

#### 3.3.4. Health Workers

Studies among healthcare workers in Nigeria have reported a wide range of HBV prevalence from 1.1% to 25.7% (median: 3.9%) [33–44]. However, differences in HBV prevalence between clinical and nonclinical staff were inconsistent across the studies [35–37, 40, 41].

### 3.4. Occult HBV Infection (OBI)

OBI refers to the presence of circulating HBV DNA in persons who test negative for HBsAg [51]. The prevalence of OBI in the general population in Nigeria has not been extensively studied [52]. However, available evidence suggests a relatively high prevalence. For instance, among 451 patients aged 1–50 years, 16.9% of those who tested negative for HBsAg were found to be HBV DNA positive [53]. Similarly, studies have reported low to high prevalence rates of OBI among blood donors (1%–17%; median: 6.7) [54–59] and people living with HIV (PLHIV) (11.2%) [60]. Of note, the measurement of OBI is inconsistent across studies, with some reporting prevalence only among HBsAg-negative and anti-HBc-positive persons [57].

### 3.5. HBV e-Antigen (HBeAg)

Studies in the general populations have reported intermediate to high prevalence rates of HBeAg ranging from 6.0% to 23.6% (median = 15%) [61–67]. A systemic review, although limited to pregnant women, reported a pooled prevalence of 14.6% [46]. Studies have also found a significantly higher rate of HBeAg in PLHIV compared with those without HIV [66, 68].

### 3.6. HBV Virus Genotypes

Evidence from studies that have conducted phylogenetic analysis of HBV in Nigeria indicates that the predominant genotype is E. studies have reported genotype E prevalence rates ranging from 44.8% to 100% (median = 97.1%) [69–77]. For example, in samples with mixed genotype infections, Ahmad et al. reported genotype E as the most predominant (97.1%), followed by genotype B (82.6%), genotype A (24.6%), genotype C (17.4%), and genotype D (0.7%) [70].

### 3.7. HBV Coinfections

#### 3.7.1. HBV and HIV Coinfection

The prevalence of HBV is high among PLHIV, with systematic reviews reporting 15% [45] and 9.9% [13]. The NAIIS also assessed HBV among PLHIV, reporting a rate of 8.9% [9]. Although slightly higher, these estimates suggest that HBV prevalence among PLHIV is comparable to that in the general population. For example, in the NAIIS data, HIV-negative individuals had a HBV prevalence of 8.1% [9].

#### 3.7.2. HBV and Hepatitis C Virus (HCV) Coinfection

There is a paucity of nationally representative data on HBV and HCV coinfection in the general population. However, findings from a national survey by Onyekwere and Hameed showed a low prevalence of 0.2% among the general population [47]. This is consistent with results from a national multicenter study among pregnant women, which reported a prevalence of 0.11% [78]. Several smaller studies involving diverse population groups have reported HBV-HCV coinfection rates ranging from 0% to 8.9% (median = 0.9%) [21, 79–88].

#### 3.7.3. HBV and Hepatitis D Virus (HDV) Coinfection

Based on anti-HDV antibody testing, a systematic review of 11 studies reported that the prevalence of hepatitis D among HBV-positive individuals ranged from 1.1% to 31.6% (median = 4.9%) [89]. Consistent with this median, a national study involving 1, 281 HBV-positive persons from Nigeria's six geopolitical zones found a prevalence of 4.8% [90]. However, data from the NAIIS indicate an intermediate prevalence of 7.3% [91].

### 3.8. Population Size Estimation of HBV

Based on earlier prevalence estimates, previous studies had extrapolated that over 20 million people are living with HBV in Nigeria [4, 5, 12]. However, recent WHO estimates indicated that approximately 14,384,770 people were living with HBV in Nigeria by the end of 2022 [3].

### 3.9. HBV Mode of Transmission

There is a lack of evidence on the predominant route of transmission of HBV Nigeria. Perinatal transmission likely accounts for the majority of infections in infants, with one study reporting a transmission rate of 42.9% among unvaccinated women [92]. Several studies have reported associations between HBV and scarifications, tattooing, circumcision, blood transfusion, sexual intercourse (multiple partnership), sharing of toothbrushes, and previous surgery [79, 93–98], suggesting possible horizontal transmission through these routes.

### 3.10. Incidence of HBV

There is a paucity of data on the new HBV infections in Nigeria. In 2022, an estimated 299,334 people were newly infected with HBV [3]. This corresponds to an incidence rate to 138 cases per 100,000 population, based on an estimated population of 216,783,381 in 2022 [99].

### 3.11. Mortality Attributable to HBV

In 2022, an estimated 46,144 deaths were attributed to HBV [3], translating to a mortality rate of 21 per 100,000 population. Estimates from an earlier GBD study indicated an increase in annual HBV-related deaths, from approximately 14,500 in 1990 to 20,200 in 2019 [14]. In contrast, age-standardized death rate declined over the same period, from 16.0 per 100,000 population in 1990 to 9.4 per 100,000 in 2019 [14].

## 4. Discussion

In this study, we reviewed and summarized the available data on the epidemiology of HBV in Nigeria. The most recent estimate indicates that Nigeria has an intermediate endemicity of HBV. This contrasts earlier estimates from systematic reviews and national surveys that classified Nigeria as a high endemic country. The decline in the prevalence of HBV may be due in part to expanded vaccine coverage. Nigeria is one of the few countries in SSA that offer universal HBV birth dose and childhood HBV vaccination [100]. Since its introduction, childhood HBV vaccine coverage has steadily increased, with reported estimates of 63% for birth dose and 52% for 3^rd^ dose by the end of 2023 [101]. Population growth and increase in HBV-related deaths may also contribute to the declining prevalence.

The results also suggest sociodemographic disparities in the prevalence of HBV, with higher rates among men, adults (> 18 years), and rural dwellers compared with their counterparts. Similar disparities related to area of residence [102, 103], sex [102, 104], and age [105] have been reported in other African countries. These disparities may be driven by differences in risk behaviors and access to healthcare. Notably, the pattern of disparity was mixed for educational attainment and economic status, highlighting the need for future studies to explore their associations with HBV.

Beyond the general population, several studies have examined the prevalence of HBV in vulnerable and high-risk populations, including healthcare workers, blood donors, and prison inmates. Despite the extensive body of literature, we did not find any national-level data or systemic reviews focused on healthcare workers. Available studies among this population report a wide range of prevalence rates, from low to high. Needle-stick injuries are likely contributors to the burden of HBV among healthcare workers. Importantly, some studies have found higher rates among nonclinical staff [35, 37, 40]. While further evidence is needed, HBV prevention and control efforts among healthcare workers should not be limited to clinical staff alone. Findings among blood donors suggest higher rates than the general population. Interestingly, family replacement donors have been reported to have higher infection rates than commercial or voluntary donors [49, 50]. The observed high prevalence of OBI among blood donors in Nigeria highlights the need for blood screening protocols with appropriate diagnostic techniques. The majority of available studies among key populations have focused on prison inmates and reported intermediate to high rates. Risk factors such as sharing sharp objects (e.g., blades and clippers) and unprotected sexual activity may contribute to transmission in prison settings [106].

Our results also underscore the burden of HBV coinfections with HIV, HCV, and HDV in Nigeria. Several studies report a high prevalence of HBV-HIV coinfection, with HBV infection rate slightly higher among PLHIV compared with the general population. In contrast, HBV-HCV and HBV-HDV coinfections appear to have low and intermediate prevalence, respectively. HBV-HDV is regarded as the most severe form of chronic viral hepatitis due to its association with more rapid progression to advanced liver disease [107]. Thus, universal anti-HDV antibody testing for HBsAg-positive persons in Nigeria may be warranted.

There is a lack of comprehensive data on the specific modes of transmission of HBV in Nigeria and their contributions to the national burden. In high-endemic settings, mother-to-child transmission is the leading mode of transmission. However, in SSA, where the prevalence of HBeAg is relatively lower in pregnant women, perinatal transmission is estimated to contribute less significantly to HBV burden [108]. HBeAg positivity indicates active replication of HBV, and it is an important factor in determining the risk of mother-to-child transmission of HBV and the likelihood of developing chronic HBV infection [109]. Notably, studies among pregnant women and general population in Nigeria report intermediate to high prevalence rates of HBeAg.

Consistent with its predominance in West Africa [110], HBV virus genotype E is the most prevalent in Nigeria. To date, at least 10 HBV virus genotypes and several subtypes have been identified, with varying geographical distribution [111]. These genotypes have clinical implications and have been linked with disease progression and treatment response [111, 112]. For example, compared with genotypes C and D, people infected with genotypes A and B tend to show a better response to interferon-based treatment. Although limited data are available on interferon treatment outcomes for genotype E [112], existing evidence suggests it may be associated with a poorer response compared with genotypes A-C [113].

The observed increasing number of HBV-related deaths is a cause for concern. Although this finding may partly reflect improved data tracking and reporting system [114], limited access to care, particularly among the aging population with chronic HBV, may also be a factor. HBV treatment coverage in Nigeria remains markedly low, estimated at 10% among those eligible [115]. HBV is responsible for about 60% of hepatocellular cancer cases in Nigeria, and the majority of the patients present at a late stage with poor prognosis [116]. To improve treatment coverage in resource-limited settings such as Nigeria, a strategy of treat-all and decentralized community treatment programs have been recommended [117]. Although Nigeria has adopted the decentralization at primary care level, treatment can only be initiated by clinicians [118], who are often in short supply at that level.

### 4.1. Limitations

The review has several limitations. The literature search was not exhaustive. We searched only four databases and prioritized larger studies with broader population representativeness. We did not perform quality assessment of the included studies, and as such, the quality of evidence may vary, with some studies likely providing low-quality data. The differences in HBV prevalence within and across populations were reported descriptively and not adjusted for potential confounding factors.

### 4.2. Recommendations for Future Research

There is a need for periodic national household surveys to monitor the prevalence of HBV in Nigeria. This could be integrated into existing platforms such as the national HIV survey or the Demographic and Health Survey. Future epidemiological studies should prioritize socially disadvantaged populations, including persons with disabilities, MSM, FSW, PWID, and transgender persons. Further research is also needed to clarify the relationship between HBV burden and socioeconomic indicators such as income and educational status. Additional evidence on the prevalence of OBI in the general population is necessary. We also recommend a systematic review and meta-analysis for a pooled prevalence rate of (i) HBeAg in the general population; (ii) HBV and HCV coinfection, and (iii) HBV among health workers. Furthermore, studies on HBV transmission dynamics and incidence are also needed to better understand drivers of the infection. Finally, spatial analyses are needed to map the geographic distribution of HBV and identify potential hotspot areas to better target interventions.

## 5. Conclusions

HBV continues to be an infectious disease of public health significance in Nigeria, with a wide range of prevalence observed across different population groups. Although recent data indicate an intermediate prevalence and declining rates in the general population, a data-driven, targeted approach to prevention and control is urgently needed to accelerate progress toward elimination. Key research gaps in HBV epidemiology that must be addressed include modes of transmission, incidence rate, prevalence among key populations, the prevalence of OBI in the general population, and spatial distribution of the infection.

## Figures and Tables

**Table 1:** Prevalence of HBV by population group.

Population	Prevalence rate^a^
General^b^	Intermediate: 5.4% [3]
	High: 8.1%–13.6% [9, 12–14]
	Median: 9.5%

Blood donor^b^	High: 13.2%–14.0% [12, 13]
	Median: 13.8%

Prison inmates	Intermediate: 4%–4.7% [15, 16]
	High: 10%–42.2% [17–24]
	Median: 15.5%

Men who have sex with men	High: 8.4%^c^–11.7% [25, 26]
	Median: 10.1%

Transgender women^c^	High: 15.6% [26]

Female sex workers	Low: 0% [27]
	Intermediate: 4% [28]
	High: 8%–17.1% [29, 30]
	Median: 6%

People who inject drugs	Intermediate: 7% [31]
	High: 7.8% [32]
	Median: 7.4%

Health workers	Low: 1.1%–1.5% [33, 34]
	Intermediate: 2.1%–7% [35–41]
	High: 13%–25.7% [42–44]
	Median: 3.9%

Pregnant women^b^	Intermediate: 5.5%–6.5% [9, 46]
	High: 7.7%–14.1% [12, 13]
	Median: 7.1%

^a^Low < 2%; intermediate: 2%–7%; high ≥ 8%.

^b^Results from systemic reviews and national surveys.

^c^Disaggregated results as provided by the authors of the study.

## Data Availability

The data that support the findings of this study are publicly available.
